# In Vitro Antioxidant and Anticancer Properties of Various *E. senegalensis* Extracts

**DOI:** 10.3390/molecules27082583

**Published:** 2022-04-16

**Authors:** Souleymane Fofana, Cédric Delporte, Rafaèle Calvo Esposito, Moussa Ouédraogo, Pierre Van Antwerpen, Innocent Pierre Guissou, Rasmané Semdé, Véronique Mathieu

**Affiliations:** 1 Laboratory of Drug Sciences, Higher Institute of Health Sciences (INSSA), Nazi BONI University, Bobo-Dioulasso 01 P.O. Box 1091, Burkina Faso; fof_soul@yahoo.fr; 2 RD3—Pharmacognosy, Bioanalysis and Drug Discovery Unit and Analytical Platform, Faculty of Pharmacy, Université Libre de Bruxelles (ULB), 1050 Brussels, Belgium; cedric.delporte@ulb.be (C.D.); pierre.van.antwerpen@ulb.be (P.V.A.); 3 Protein Chemistry Unit, Department of General Chemistry I, Faculty of Medicine, Université Libre de Bruxelles, Campus Erasme (CP 609), Route de Lennik, 1070 Brussels, Belgium; rafaele.calvo.esposito@ulb.be; 4 Department of Pharmacotherapy and Pharmaceuticals, Faculty of Pharmacy, Université Libre de Bruxelles (ULB), 1050 Brussels, Belgium; 5 Laboratory of Drug Development (LADME), Center of Training, Research and Expertises of Pharmaceutical Sciences (CEA-CFOREM), Training and Research Unit, Health Sciences, Joseph KI-ZERBO University, Ouagadougou 03 P.O. Box 7021, Burkina Faso; ouemoussa10@gmail.com (M.O.); rsemde@yahoo.fr (R.S.); 6 Faculty of Health Sciences, Saint Thomas d’Aquin University, Ouagadougou 06 P.O. Box 10212, Burkina Faso; ip_guissou@yahoo.fr; 7 ULB Cancer Research Center, Université libre de Bruxelles (ULB), 1050 Brussels, Belgium

**Keywords:** *E. senegalensis*, cytotoxicity, paraptosis, ROS, vacuoles, anticancer, antioxidant, stigmastane steroid, senegalensein

## Abstract

Although *Erythrina senegalensis* is a plant widely used in traditional medicine in sub-Saharan Africa, its biological properties have been poorly investigated to date. We first characterized by conventional reactions the composition of several stem bark extracts and evaluated in acellular and cellular assays their pro- or antioxidant properties supported by their high phenolic and flavonoid content, particularly with the methanolic extract. The pro- or antioxidant effects observed did not correlate with their IC_50_ concentrations against five cancer cell lines determined by MTT assay. Indeed, the CH_2_Cl_2_ extract and its ethyl acetate (EtOAc) subfraction appeared more potent although they harbored lower pro- or antioxidant effects. Nevertheless, at equipotent concentration, both extracts induced ER- and mitochondria-derived vacuoles observed by fluorescent microscopy that further led to non-apoptotic cell death. LC coupled to high resolution MS investigations have been performed to identify chemical compounds of the extracts. These investigations highlighted the presence of compounds formerly isolated from *E. senegalensis* including senegalensein that could be retrieved only in the EtOAc subfraction but also thirteen other compounds, such as 16:3-Glc-stigmasterol and hexadecanoic acid, whose anticancer properties have been previously reported. Nineteen other compounds remain to be identified. In conclusion, *E. senegalensis* appeared rich in compounds with antioxidant and anticancer properties, supporting its use in traditional practice and its status as a species of interest for further investigations in anticancer drug research.

## 1. Introduction

Cancer is one of the leading causes of death worldwide, accounting for nearly 10 million deaths in 2020. Seventy percent of them occurred in low- and middle-income countries due to late-stage discoveries, diagnosis inability and poor access to appropriate treatment [1]. Plants have been and are still widely used in traditional medicine all over the world, being a historical source of drugs. Moreover, between 1981 and 2014, a significant proportion of drugs approved by the FDA were developed or synthesized from natural products such as plants [2]. More precisely, among all anticancer drugs developed between 1940 and 2014, 12% of them are natural products, 25% are derivatives of natural products and 9% are synthetic molecules mimicking the activity of natural substances [2]. Thus, about 46% of anticancer drugs are directly derived from or inspired by nature. Despite the major advances in cancer chemotherapies, which are adapted to each type of cancer, these treatments may provoke major side effects, since they target cell division in a non-selective way, thereby having collateral effects on healthy cells as well. To overcome this problem, research aiming at identifying new natural substances that may be used alone or in combination with current treatments is steadily increasing. Indeed, numerous plant-derived substances are endowed with anticancer activity targeting proteins specially deregulated in cancer [3], thus activating non-apoptotic cell death as an alternative to dysregulated apoptosis. In addition, other plant-derived substances may improve the tolerance of several chemotherapies [4,5]. 

A systematic review carried out by Bayala et al. [6] on anticancer plants and the works of Sawadogo et al. [7,8] showed that the flora of Burkina Faso (West Africa, Sahel) is rich in potential anticancer substances. *Erythrina senegalensis* is a medicinal plant used in traditional medicine in sub-Saharan Africa (Mali, Burkina Faso, Nigeria, Cameroon) for the treatment of numerous diseases. Despite the variability among the healers regarding its medicinal uses, ethnopharmacological studies conducted in three regions of Mali reported malaria, jaundice, infections, digestive disorders, pain, weakness and gyneco-obstetrical troubles as the main diseases/ symptoms against which *E. senegalensis* extracts are used [9]. Whether extracts of *E. senegalensis* are used in the traditional treatment of cancers remains poorly documented, probably because African traditional practitioners often treat symptoms rather than diseases. Noteworthy, jaundice, pain and weakness are frequent symptoms of advanced cancer patients. 

In addition, scientific consistency of all medicinal uses is supported by numerous studies highlighting the anticancer [10], antihypertensive, antidiabetic [11], antiinflammatory, antiplasmodial [12], antibacterial [13,14], anti-HIV [15], antiparasitic [16], antioxidant [17,18] and enzyme inhibitory [19,20] properties of extracts and/or secondary metabolites isolated from this plant. Similar broad range of pharmacological activities has been observed with respect to the *Mimosa* genus [21,22].

**Figure 1:**
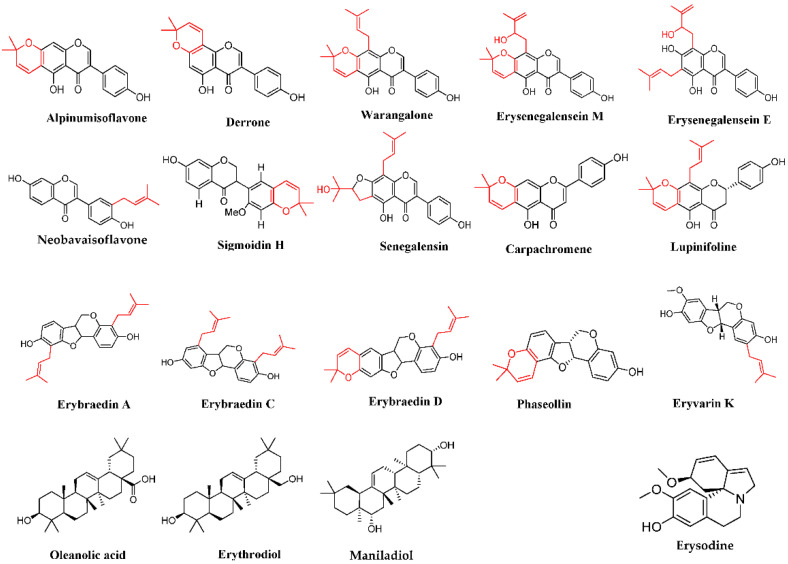
Chemical structures of compounds known in *E. Senegalensis* with antioxidant and/or in vitro and in vivo anticancer effects. Erybraedin D and eryvarin K are antioxidant by inhibiting 12-lipoxygenase without demonstrated antiradical or cytotoxic activity [19]. Alpinumisoflavone, carpachromene, lupinifolin, oleanolic acid and erythrodiol are both antioxidant and cytotoxic [26,44,45,46,47,48,49,50,51,52,53]. The other secondary metabolites have only demonstrated cytotoxic activity.

Among the compounds isolated from *E. senegalensis* to date harboring anticancer effects in vitro or in vivo, most of them are prenylated flavonoids or isoflavonoids and triterpenes [23] (Figure 1). The anticancer effects’ mechanisms were at least partly deciphered for oleanolic acid [24,25], erythrodiol [26], alpinumisoflavone [27,28,29,30], derrone [31,32], warangalone [33], erybraedins A [34], C [35,36] and phaseolin [37] that generally trigger apoptosis. Alpinumisoflavone was also shown to trigger pyroptosis [29], while derrone and oleanolic acid can induce autophagy [32,38,39,40]. In addition, carpachromene, neobavaisoflavone, sigmoidin H, maniladiol and erysenegalenseins E, M are endowed with antiproliferative activity [10,41,42,43].

Although cancer initiation and pathogenesis may be potentiated or aggravated by oxidative stress [54,55,56] numerous anticancer agents trigger severe oxidative stress and damage in cancer cells, leading to cell death [57]. Considering the 42 known compounds in *E. senegalensis* identified to date [23], the probability that *E. Senegalensis* extracts may have an impact on the cellular oxidative balance is high. Accordingly, total extracts of the plant were excellent scavengers of free radicals as well as inhibitors of oxidative enzymes such as lipoxygenase [19,58]. However, the antioxidant activity of its secondary metabolites remains poorly studied, except in the work of Togola et al. 2009 evaluating the lipoxygenase inhibitory activity of erybraedins A, C, D, phaseollin and eryvarin K [19] as well as in other works conducted on alpinumisoflavone [45,46,47], carpachromene [49] and oleanolic acid [48]. Oxidative stress plays also key roles in bacterial, viral and parasitic infections [59], including malaria [60], all of which are treated by traditional healers with *E. senegalensis* extracts [9,61].

This work aimed, therefore, to investigate both the in vitro antioxidant and anticancer properties of various *E. senegalensis* extracts phytochemically characterized by conventional reactivity assays and by liquid chromatography coupled to high resolution mass spectrometry (LC-MS).

## 2. Results

### 2.1. Extracts Preparation and Their Chemical Class Composition

The stem bark of *E. senegalensis* DC (Fabaceae) was the starting material of the present study. The residual moisture content of the analyzed powder was estimated at 5.3% after air drying at 120 °C. Extraction with a dichloromethane/methanol mixture (CH_2_Cl_2_/MeOH 1:1) had given the best overall massic yield, i.e., 10.2% followed by extraction with methanol (MeOH) (9.1%) and dichloromethane (CH_2_Cl_2_) (5.9%).

Conventional reagents for determination of the presence of main chemical families were first used. The main phytochemical groups present in the stem bark of E. senegalensis were flavonoids, tannins, coumarins, saponosides, emodols, anthracenosides, triterpenes and sterols (Table 1). Thus, this study reveals for the first time, at least to our best knowledge, the presence of tannins, saponosides, emodoles, anthracenosides, coumarins and derivatives in the extracts of *E. senegalensis* [10,19,62,63,64,65,66,67,68,69,70]. Importantly, these chemical families include numerous compounds with antioxidant and/or anticancer properties [71,72,73,74,75,76,77,78,79,80]. Reactions to detect alkaloids, anthocyanosides and cardiotonic heterosides were negative for the three extracts of *E. senegalensis*. Unsurprisingly, the CH_2_Cl_2_/MeOH (1:1) snippet seemed to contain both major chemical groups found in either CH_2_Cl_2_ or MeOH snippets.

### 2.2. In Vitro Growth Inhibitory Effects of the Extracts Assessed by MTT Assay

The antiproliferative effects of each extract was then evaluated using the colorimetric MTT assay on five cancer cell lines, one of them being of murine origin, i.e., B16F10, while the other four are of human origin. We observed that the IC_50_ of the primary extracts varied from 19 to 77 µg/mL depending on the extract solvent and the cell line used (Table 2). Importantly, we noticed that although the CH_2_Cl_2_/MeOH extract contained both kinds of chemicals found in the CH_2_Cl_2_ extract and the MeOH extract, respectively, its IC_50_ was not better than the one of the methanolic extract. The CH_2_Cl_2_ extract appeared actually the most promising one with a mean IC_50_ of 30 µg/mL. Re-extraction of this extract was, thus, conducted by successive percolation using solvents of increasing polarity (n-hexane, ethyl acetate, acetone) in an open chromatography column. The n-hexane subfraction was insoluble in culture medium, while the recovery of the acetone subfraction after filtering silica did not yield sufficient materials for characterization and testing.

Notably, the EtOAc subfraction retained the activity of the CH_2_Cl_2_ extract (Table 2). The conventional characterization of this subfraction revealed flavonoids (+), sterols and triterpenes (+) and coumarins (+).

### 2.3. Antioxidant Effects of the Extracts from E. senegalensis

We next evaluated the total phenolic and flavonoid content of each fraction (Table 3). MeOH extract displayed the highest phenolic and flavonoid contents. In contrast, the EtOAc subfraction contained the lowest levels of gallic acid (GA) and quercetin (Q) equivalents (see Table 3). Their antioxidant effects were evaluated through DPPH, ABTS and FRAP scavenging assays. Despite its lower proportion of GAE (62%) and QE (47%), the CH_2_Cl_2_/MeOH extract harbored similar antioxidant activities to the MeOH extract. By contrast, the CH_2_Cl_2_ extract and its EtOAc subfraction displayed similar antioxidant activities but those later were markedly lower than those of the other extracts. Thus, the use of MeOH appeared to play a key role in the extraction of potent antioxidant compounds, being accordingly the most common solvent used for this purpose [10,19].

**Figure 2:**
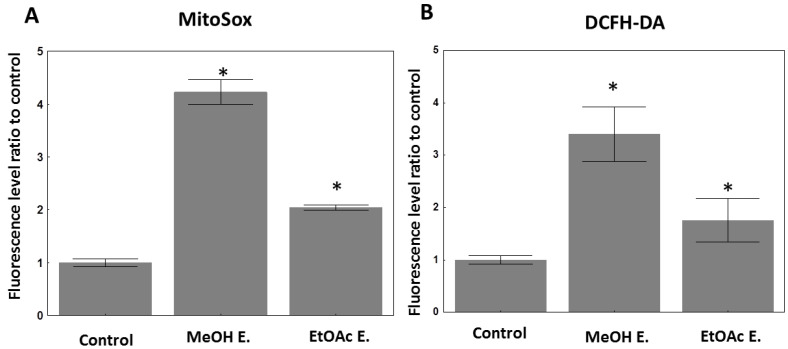
ROS levels in U373 cells studied by flow cytometry. (**A**): MitoSox staining: mean fluorescence intensity of 10,000 cells was registered. The mean fluorescence level of the untreated cells was normalized to 1. (**B**): DCFH-DA staining data (same legend). Data are presented as mean +/− SEM of triplicates. MeOH E.: methanol extract; EtOAc E.: ethyl acetate subfraction. MeOH extract was applied at 70 µg/mL, while EtOAc at 40 µg/mL during 40 h on the cells before staining. * means *p* < 0.05 in comparison to the control group.

We further compared the effects of MeOH extract to the EtOAc one on the total levels of reactive oxygen species (ROS; DCFH-DA) as well as the mitochondrial ROS (MitoSox) in one cellular model, i.e., the U373 cell line through flow cytometry. Results highlight that although both extracts induced a strong increase in total ROS, the effects were much more pronounced with the MeOH extract at their equipotent IC_50_ concentration (Table 2; Figure 2). This increase seemed to originate from the mitochondria according to the MitoSox staining (Figure 2). MeOH extract contains about five times more phenolic and flavonoids than the EtOAc extract, supporting the theory that these kinds of molecules contributed at least partly to this effect. The increase in cellular ROS induced by those extracts while they display in vitro antioxidant effects in an acellular context is not surprising. Indeed, numerous phenolic and flavonoid compounds are known to play either antioxidant or pro-oxidant roles depending on the cell types and their cellular context [81,82], the most famous example being curcumin [83].

### 2.4. E. senegalensis Extracts Induce Morphological Changes including Vacuolization of Cancer Cells

Altogether, these data suggest that the potency of the in vitro anticancer properties of the extracts does not correlate to the induction of ROS imbalance. We decided, therefore, to further investigate their effects by phase contrast microscopy.

**Figure 3:**
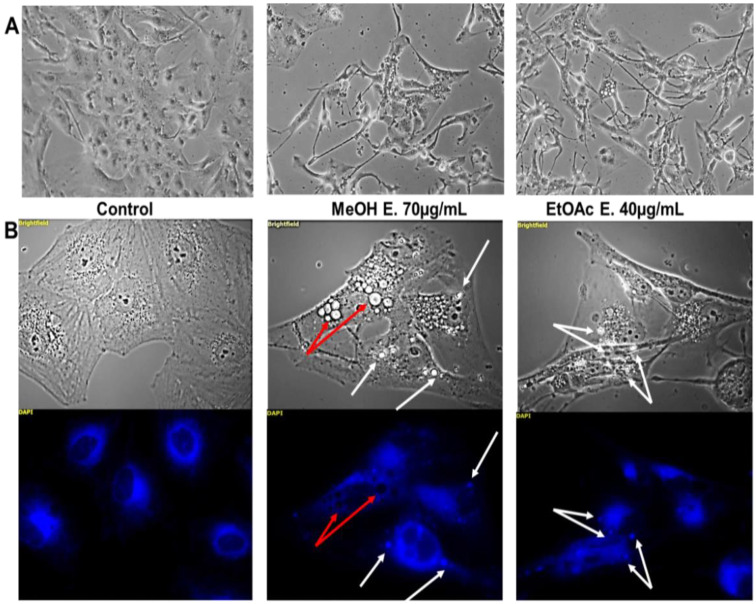
Morphological changes and vacuolization induced by *E. senegalensis* extracts. (**A**): phase contrast micrographs of U373 cells treated or not treated with the extracts for 40 h (GX100). (**B**): Brightfield and fluorescent pictures of U373 cells stained with ER-Tracker^®^ after 40 h of treatment. White arrows highlight ER-derived vacuoles, while red arrows point other kinds of vacuoles (GX400).

Strong morphological changes were observed after 40 h of treatment with both extracts used at their own IC_50_ (Figure 3A). Cells seemed to be still viable, adherent but with modified shape (thinner, elongated and star-like morphology) and bearing increased vacuolization processes. The origin of the latter processes was investigated through fluorescent microscopy and flow cytometry. The autophagic phenomenon, which is known to trigger vacuolization process [84], was ruled out according to the absence of increase in red acridine orange staining expected in case of autophagy and the absence of Lysotracker^®^ staining in the vacuoles (data not shown). We next envisaged that these vacuoles may arise from the mitochondria and/or the endoplasmic reticulum. The ER-tracker^®^ strongly stained several bright vacuoles (Figure 3B; white arrows). Notably, the signal was not attributed to the fluorescence of one or several compounds of the extracts, since unstained but treated cells did not display such a strong signal (data not shown).

**Figure 4:**
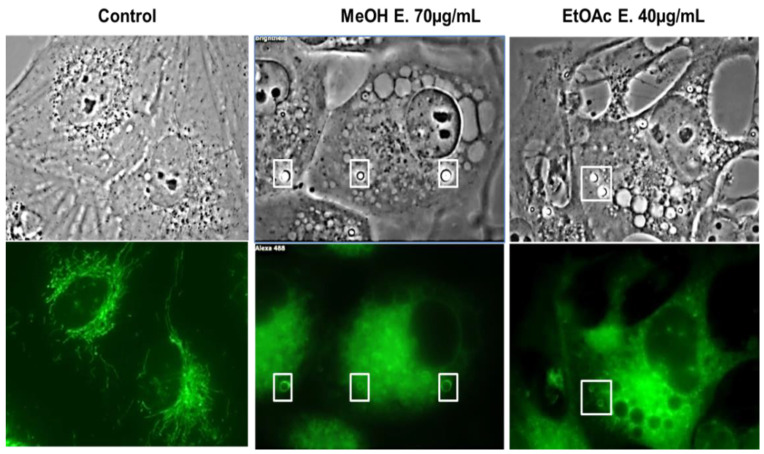
Mitochondrial network study. Brightfield and fluorescent pictures of U373 cells stained with Mito-Tracker^®^ after 48 h of treatment. A normal mitochondrial network is observed in untreated cells. Treated cells harbor disrupted network in addition to mitochondrial-derived vacuoles whose membrane is positively stained (examples highlighted in the white squares; GX1000).

In addition, the mitochondrial network appeared strongly affected as observed on high magnification captions (Figure 4). Several bright vacuoles (white squares) displayed strong membrane staining with the MitoTracker^®^. This is particularly the case in the cells treated with the methanolic extract. These mitochondrial network damages are in accordance with the strong increase in mitochondrial ROS levels observed above, notably with the MeOH extract. Several vacuoles however remained unstained by both dyes (Figure 3B; red arrows), and further investigations should be pursued to decipher their origin.

**Figure 5:**
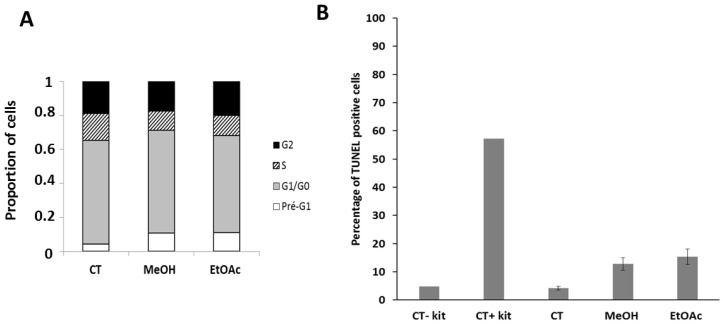
Effects of the extracts on cell cycle (**A**) and apoptosis (**B**) in U373 cells. All the experiments were carried out in triplicate and the results expressed as a mean ± SEM. After 48 h of treatment with the MeOH extract or the EtOAc sub-fraction, the distribution of cells per phase was comparable to that of the control (CT U373), and the percentage of cells in apoptosis was low, hardly exceeding 15%.

Vacuoles originating from the ER and the mitochondria have been observed in paraptosis-like phenomenon [85]. Accordingly, apoptosis is not likely induced by these extracts, when considering the TUNEL staining in this cell line (Figure 5B). Furthermore, no clear cell cycle blockage could be observed (Figure 5A).

In conclusion, it appears that several cellular effects are triggered by either kind of extracts, (i.e., the methanolic one and the EtOAc subfraction of the CH_2_Cl_2_ extract), such as modification in shape and cytoplasmic vacuolization. The stronger ROS imbalance, particularly at the mitochondrial level, observed with the methanolic extract might be linked to its higher flavonoid and phenolic content. To better understand the similarities and differences observed between these two extracts, we conducted preparative TLC followed by LC-MS to characterize their composition as best as possible.

### 2.5. Identification of Substances of E. senegalensis Extracts by LC-HRMS

Interestingly, our data supported the presence of 13 known compounds, such as 2,4,6-trihydroxychalcone, phytosterols and several triterpenes: uvaol, 7-campestenol, 22-hydroxycampesterol and ergostanol, which were never identified in *E. Senegalensis* (Table 4, Appendix A), at least to our best knowledge, in addition to eight metabolites formerly isolated from *E. senegalensis:* alpinumisoflavone (or derrone), auriculatin (or auriculasin), 2,3 -dihydroauriculatin, sigmoidin H, senegalensein, erythrisenegalone, erysenegalensein N and erythrinasinate (Table 5, Appendix B). Senegalensein was only retrieved in the EtOAc extract, while the others could be detected in both extracts.

In particular, we noticed that both extracts contained compounds determined by the program as 7-campestenol, 9,12-octadecadienoic acid, α-linolinic acid, isofucosterol, 3-O-[6-O -hexadecanoyl-b-D-glucopyranoside], 2,3-dihydro-auriculatin, alpinumisoflavone (or derrone), erythrisenegalone and sigmoidin H (Table 4). Although our method does not allow us to exclude the presence of one or another metabolite when no corresponding signal was retrieved, we may hypothesize that MeOH extract could be enriched in 1,4-benzenediol (hydroquinone), 2′,4′,6′-trihydroxy chalcone, ergostanol, uvaol, auriculatin (or auriculasin), erysenegalensein N and erythrinasinate in comparison to the EtOAc subfraction. Reversely, the EtOAc subfraction may be enriched in 16:3-Glc-stigmasterol, 22-hydroxy-campesterol, hexadecanoic acid, feruloyldihydro-beta-sitosterol and senegalensein.

In addition, we found 19 other compounds whose structure identification or elucidation is still required (Table 6). Work to that aim is still ongoing.

## 3. Discussion

In general, the MeOH extract displayed moderate cytotoxic activity, while the EtOAc sub-fraction, derived from the CH_2_Cl_2_ extract, was the most active against the five cell lines used in this study. To investigate the chemical composition of the extracts, we used LC-HRMS, a highly used methods for metabolite identification in plant extracts. Both extracts contain substances whose cytotoxic effects are well demonstrated such as erythrisenegalone, alpinumisoflavone, auriculatin (or auriculasin) and sigmoidin H [10,27,29,31,32,86,87,88,89,90]. According to the known low to moderate potency of these compounds [23] and their limited abundance within the extracts, other compounds newly identified in *E. senegalensis* in this study might contribute significantly to their anticancer properties. MeOH extract contained 1,4-benzenediol (hydroquinone), 2′,4′,6′-trihydroxy chalcone, ergostanol and uvaol, in comparison to the EtOAc subfraction, which might be enriched in 16:3-Glc-stigmasterol, 22-hydroxy-campesterol, hexadecanoic acid and feruloyldihydro-beta-sitosterol.

The cytotoxic effects of 1,4-benzenediol [91,92,93], stigmasterol derivatives [94,95,96] and 9,12-octadecadienoic acid (Z, Z) [94] were reported previously. The anticancer properties of fucosterol derivatives studied in vitro in various cancer cell types [97,98,99,100] seem to rely on apoptosis induction, at least in Hela cell model by inhibiting the PI3K/AKT cascade [100]. Stigmastane-type steroids can trigger cell cycle arrest in the G2/M or G0/G1 phase, leading to intrinsic apoptosis [101,102]. Uvaol also activates apoptosis in many cellular models, consequently leading to cell cycle arrest in the G0/G1 phase, production of ROS or affecting the AKT/PI3K signaling pathway [52,53,103,104].

In the present study, cells treated with our extracts displayed strong morphological changes associated with mitochondrial network disruption and vacuolization arising from both the ER and the mitochondria. Those features are suggestive of paraptosis, a caspase-independent programmed cell death that differs morphologically and biochemically from apoptosis [105]. Ultrastructurally, cells lack the features of apoptosis, such as nuclear fragmentation, formation of apoptotic bodies and condensation of chromatin. Instead, vacuolization in the cytoplasm originates primarily from the endoplasmic reticulum (ER) and is accompanied by swelling and agglutination of the mitochondria as well as a collapse of the cytoskeleton prior to cell death [105]. Although many of the substances newly identified in *E. Senegalensis* in the present study are known to be cytotoxic, none of these were yet shown to be able to trigger paraptosis. Interestingly, paraptosis may be linked to increase in ROS and ER stress [106], as observed in the present study with both extracts in U373 cells. However, the MeOH extract induced stronger increase in ROS at equipotent concentration than the EtOAc subfraction. Other kind of effects induced by other chemicals of different classes, including those found for the first time in this study in *E. Senegalensis*, might, thus, contribute significantly to the higher in vitro anticancer potency of the EtOAc subfraction. The search for pharmacological agents capable of inducing non-apoptotic death in cancer cells is very promising, because most current anticancer drugs are pro-apoptotic, and resistance to apoptosis may lead to treatment failure [107]. In particular, some compounds, such as the polyphenol curcumin, have been found to induce paraptosis-like cell death in apoptosis-resistant cancer cells [77,79]. This may explain why many molecules targeting paraptosis are actively studied to improve cancer therapy [108].

In addition to cancers, ROS imbalance has been involved in the development of numerous other pathologies, including neurological disorders (Alzheimer’s and Parkinson’s diseases and amyotrophic lateral sclerosis) [109], cardiovascular diseases, chronic obstructive pulmonary disease, asthma, rheumatoid and osteoarthritis, among others [56,109,110,111,112,113]. Whether the antioxidant effects of our extracts could be of potential interest in the prevention or in the reduction of the pathogenesis of those diseases warrants further investigation too. This is particularly the case with respect to the methanolic extract, which was the richest extract in phenolic content and accordingly harbored the best in vitro antioxidant effects in acellular assays (10 times more potent than the EtOAc subfraction). This study showed once again the double face of polyphenols: commonly considered as antioxidant agents, they might turn into pro-oxidant ones according to the metabolic status of the cell, opening the way to multiple potential medical applications.

## 4. Materials and Methods

### 4.1. Chemistry

#### 4.1.1. Plant Material

The stem bark of Erythrina senegalensis DC (Fabaceae) was harvested in January 2017 at 17 km (UTM: X = 30P03434582; Y = 1239658) from Bobo-Dioulasso (Burkina Faso). After authentication by an expert in botany (GANABA Souleymane, National Center for Scientific and Technological Research), a specimen was deposited at the National Herbarium of Burkina (HNBU n° 8709). The herbal drug was dried in the open air out of direct sunlight for 10 days and then ground into a coarse powder. The residual moisture content (RMC) of this powder was determined according to the official thermogravimetric method AOAC 925.10 (AOAC, 1990) based on the removal of water from the sample by heating in a ventilated oven. Three samples of 5.00 g of herbal drug were placed in a ventilated oven at 105 °C for 3 h. The crucibles were then removed, cooled in a desiccator for 30 min and weighed. The operation was repeated until the dried mass remained unchanged. The THR of the analyzed sample was calculated according to the following formula:

(1) $$THR\left(\%\right)=\frac{Pe-Pe\prime }{Pe}\times 100$$

where *Pe* = the precise mass of the test sample (g) and *Pe’* = the precise mass of the test sample after drying (g).

#### 4.1.2. Reagents and Solvents

Solvents used in this study for extractions are dichloromethane (Carlo Erba, Paris, France), methanol (Merk, Darmstadt, Germany) and ethyl acetate (VWR, Prolabo, Paris, France). For LC-MS analyses, acetonitrile and formic acid was of LC-MS quality (Fisher Scientific, Bruxelles, Belgium)

Reagents used in this study are as follows: Folin–Ciocalteu reagent, sodium acetate trihydrate (Sigma–Aldrich, Berlin, Germany), 1,1-diphenyl-2-picryl hydrazyl (DPPH), 2,2′-azino-bis (3-ethylbenzothiazoline-6-sulfonic acid) diammonium salt (ABTS), potassium persulfate (di-potassium perox-disulfate), cyanidin 3-glucoside, alizarin red, 6-hydroxy-2,5,7,8-tetramethylchroman-2-carboxylic acid (Trolox), gallic acid and quercetin were purchased from Sigma Aldrich (Germany); Iron (II) sulfate heptahydrate was obtained from Acros Organics (Fisher Scientific, London, UK); and 2,4,6-tripyridyl-s-triazine (TPTZ) from Fluka U.K. Sodium carbonate (Na_2_CO_3_) and aluminum trichloride (AlCl_3_) were purchased from Carlo Erba (Paris, France).

#### 4.1.3. Extraction Method

The extracts were obtained from a 24 h maceration of 500 g (250 g × 2) of dried plant material under continuous stirring in a total volume of 2.5 L of solvent (MeOH, CH_2_Cl_2_ or CH_2_Cl_2_/MeOH 1:1). The three different extracts were then percolated with small volumes of the same solvent until exhaustion. The three percolates obtained were concentrated under reduced pressure with a rotary evaporator (ROTAVAPOR BUCHI^®^ RE 11). The MeOH and CH_2_Cl_2_/MeOH concentrated percolates were then frozen at −12 °C and lyophilized under high vacuum at −52 °C (Christ Alpha 1-2 LD plus, Germany series 19971). The extraction yield was determined by relating the mass of the dried extract obtained over the mass of herbal drug used and was expressed per 100 g of dried material.

#### 4.1.4. Phytochemicals Characterization

The chemical characterization was adapted from the method described by Ciulei [114]. Sterols and triterpenes were sought according to the Liebermann–Burchard (H_2_SO_4_ conc) reaction, alkaloids using the Dragendorff (bismith nitrate K^+^ iodide) and Mayer (Mercury chloride K^+^ iodide) reagents, anthracenics according to the Bornträger (NH_4_OH 25%) reaction, coumarins and derivatives according to the Feigl-Frehden-Anger reaction, flavonoids according to the Shibata reaction (Cyanidin test) and anthocyanosides using sodium hydroxide tablet. The tannins were characterized using Stiasny reactif (Formol 40% m/v + HCl 1N 1:1) completed with ferric chloride (FeCl_3_) at 2% in alcoholic solution. The presence of saponosides in the extracts was highlighted by the foam index, which corresponds to the height of a column of foam formed (minimum 1 cm) that persists for 15 min after vigorous stirring for 15 min.

The Folin–Ciocalteu method was used to determine total phenolic content as described by Meda et al. [115] with slight modifications. Briefly, the extract was first solubilized in methanol at 1.0 mg/mL and further diluted 10 times in distilled water. Then, 0.125 mL of this working solution was mixed with 0.625 mL of 0.2 N Folin–Ciocalteu reagent for 5 min and then with 0.5 mL of 75 g/L sodium carbonate (Na_2_CO_3_). After 2 h of incubation at room temperature, the absorbance of the reaction mixture was measured at 760 nm. A gallic acid (0 to 200 mg/L) standard calibration curve was used to graphically determine the concentration of total phenolic in the extract ($y=4.668\cdot 10^{-3}x-0.034,\;r^{2}=0.9991)$. Results are expressed in mg of Gallic Acid Equivalent per 100 mg of the extract (mg GAE/100 mg).

The total flavonoid content was determined using the Dowd method as adapted by Arvouet-Grand et al. [116]. The total flavonoid content was determined using a standard curve of quercetin ($y=1.259\cdot 10^{-2}x,\;r^{2}=0.9990$) from a range of concentrations from 0 to 50 mg/L. The results were expressed in mg of Quercetin Equivalent per 100 mg of the extract (mg QE/100 mg).

#### 4.1.5. Fractionation Method

The CH_2_Cl_2_ extract (14 g) was fractionated in an open chromatography column on silica gel (140 g) Merck Kieselgel G60; 0.2–0.5 mm (35–70 mesh ASTM) with successive elutions until the percolates are clear using solvents of increasing polarity (n-hexane, ethyl acetate and acetone). One hour contact with each of the solvents preceded percolation. Three primary fractions were obtained: n-hexane (n-Hex), ethyl acetate (EtOAc) and acetone (AcEt).

#### 4.1.6. LC-MS Process, Data Acquisition and Analysis

To improve the detection of their components by LC-MS, the MeOH, CH_2_Cl_2_ and EtOAc extracts were first fractionated by preparative TLC (pTLC) S. The plates used were of the MERCK type (glass support 20 × 20 cm; silica gel G60 F254; thickness 1 mm). A strip of approximately 2 mL of each extract was eluted with the toluene/ethyl acetate (7:3) nonpolar solvent system for 60 min. After migration over a path of 18 cm, the plates were dried on the bench and then observed under a UV lamp at 254 nm and 366 nm and the chromatograms photographed. Subsequently, all spots in migration bands were scraped off and solubilized in small volume of ethyl acetate. After filtration through a 0.45 μm nylon millipore filter and evaporation of ethyl acetate, the dry powders of the subfractions were collected in colored (brown) 2 mL vials. A homemade database was checked and completed with information specific to substances already known and isolated from the *E. senegalensis* (exact name, molecular mass and *m*/*z* ratio). Conveniently, a volume of 10.0 μL of the sample (2.0 mg of dry extract + 190 μL MeOH + 10 μL of formic acid) was automatically injected into the LC system. Analyses were performed with rapid resolution LC (RRLC) 1200 series from Agilent Technologies (Santa Clara, CA, USA). The separation was carried out in a phase gradient with water supplemented with 0.1% formic acid/acetonitrile in a chromatographic column of the Zorbax Eclipse XDB-C18 Rapid Resolution HT column (4.6 × 50 mm, 1.8 μm; Agilent Technologies) preceded by a Zorbax Eclipse XDB-C18 pre-column (4.6 × 5 mm, 1.8 μm). MS analyzes were performed using an ESI-Q-TOF (6520 series, Agilent Technologies), the parameters of which were set as follows: positive mode; capillary voltage at −4000 V; high dynamic resolution acquisition mode (2 GHz); gas temperature at 350 °C with a flow rate of 9 L/ min; nebulizer pressure at 45 psi; the fragmentor voltage at 130 V; and the skimmer voltage at 65 V. Data acquisition was possible using the Mass Hunter Acquisition software (Agilent Technologies, version B.04 SP3, Diegem, Belgium) and their analysis was made by using the Mass Hunter Quantitative Analysis software (Agilent Technologies, version B.07 SP1). The compounds were extracted by molecular feature extractor and each compound characterized by their exact mass and isotopic profile was identified according to the database.

### 4.2. Activity Assays

#### 4.2.1. Antioxidant Activity Evaluation

The DPPH assay was adapted from Xiao et al. [117]. Briefly, the reaction mixture contained the sample (extract or trolox used as positive control) in dimethyl sulfoxide and DPPH (1,1-diphenyl-2-picryl hydrazyl) at 0.2 mM in methanol. The reaction mixture was incubated at 37 °C for 30 min. The absorbance was measured at 517 nm. The radical scavenging activity was determined by comparison with a DMSO-containing control. Inhibitory concentration 50 (IC_50_) values represent the concentrations of each sample allowing scavenging of 50% of DPPH radicals. Results are expressed as Trolox equivalent antioxidant capacity (TEAC) calculated as follows: TEAC = IC_50_ of Trolox (μg/mL)/IC_50_ of the sample (µg/mL). The higher the TEAC value, the higher the DPPH radical scavenging activity [117].

The ABTS assay was conducted as follows. ABTS cation radicals were first prepared according to Nenandis et al. [118]. Then, 5 mL of aqueous ABTS solution (7 mM) was mixed with 88 μL of 140 mM K_2_S_2_O_8_. The mixture was incubated in the dark for 16 h and diluted with methanol until the absorbance value at 734 nm reached 0.7. Then, 2940.0 µL of the prepared ABTS cation radical solution were mixed with 60.0 µL of extracts solubilized in methanol and vigorously shaken for 30 s before measurement of the absorbance at 734 nm. 

The Ferric reducing antioxidant power (FRAP) assay was performed as described by Benzie and Strain [119]. The FRAP reagent was prepared by mixing 25 mL of 300 mM acetate buffer, 2.5 mL TPTZ in 40 mM HCl and 2.5 mL of 20 mM FeCl_3_.6H_2_O, at a proportion of 10:1:1 at 37 °C. Freshly prepared working FRAP reagent (3.995 mL) was mixed with 5.0 μL of the appropriately diluted plant sample and mixed thoroughly. After 30 min of incubation at 37 °C, the absorbance was measured at 593 nm against a blank (3.995 mL FRAP reagent + 5.0 μL distilled water). For both assays, Trolox was used as the standard and distilled water as the blank control. Results are expressed as µmol TE/g DW according to the formula: ABTS versus FRAP value (μmol TE/g DW) = c × V × t/m, where “c” is the Trolox concentration (μmol/mL) of the corresponding standard curve of the diluted sample, “V” is the sample volume (mL), “t” is the dilution factor and “m” is the weight of the sample dry matter (g) [120].

All the determinations were performed in triplicates.

#### 4.2.2. Culture Media and Cancer Cell Lines

The cancer cell lines used in this study were obtained from the American Type Culture Collection (Manassas, VA, USA) or from the European Collection of Cell Culture as follows: the human glioma U373 cell line (ECACC, code 08061901), two melanoma cell lines: the human SKMEL-28 (ATCC code HTB 72) and the murine B16F10 cell lines (ATCC code CRL-6475) and two carcinomas: breast MCF-7 (code ATCC, HTB-22) and NSCLC A549 (DSMZ code ACC107) cell lines. All cells were cultivated in RPMI 1640 culture medium (Gibco, Thermofisher, Dilbeek, Belgium), enriched with 10% heat-inactivated fetal bovine serum, 0.6 mg/mL of glutamine (GibcoBRL, Invitrogen, Merelbeke, Belgium), 200 IU/mL of a mixture of penicillin and streptomycin (GibcoBRL) and 0.1 mg/mL of gentamicin (GibcoBRL).

#### 4.2.3. MTT Colorimetric Assay

The effect of the extracts on overall cell growth was evaluated using the MTT (3 [4,5-dimethylthiazol-2yl] -diphenyltetrazolium-bromide) colorimetric test (Sigma-Aldrich) as previously described [121].

Briefly, 12,000 to 25,000 cells/mL depending on the cell line were seeded in 96-well plates and incubated for 24 h before applying the treatment with the extracts of *E. senegalensis*. Each experimental condition was analyzed in sextuplicate, with nine concentrations ranging from 100 μg/mL to 0.01 μg/mL. Three independent experiments were carried out in sextuplicate each.

#### 4.2.4. Phase Contrast Microscopy

Human U373 glioblastoma cells were seeded in 6-well plates and allowed adhering and growing to 60–70% before their treatment (or left untreated) with the extracts for 40 h in duplicates. Micrographs were taken with classic phase contrast microscope coupled with an Olympus camera. Representative pictures are provided for global morphological evaluation of the effects of the extracts.

#### 4.2.5. Fluorescent Microscopy for Vacuole Characterization

Evaluation of the origin of the vacuoles observed in treated cells by phase contrast microscopy was carried out using various fluorescent probes, i.e., LysoTracker^®^, ER-Tracker^®^ and MitoTracker^®^ (Molecular Probes, Life Technologies (Merelbeke, Belgium), as described by Colin et al. [121]. Briefly, U373 cells were seeded on glass coverslips placed in 6-well plates (Sarstedt) at least 24 h prior to the experiment to allow them to adhere and grow. Cells were then treated with the MeOH extract (70 μg/mL) or the EtOAc subfraction (40 μg/mL). During the last hour of treatment, the probes were added to the culture medium as follows: LysoTracker^®^ (75 nM), MitoTracker^®^ (300 nM) or ER-Tracker^®^ (0.5 µM). At the end of the incubation period, the coverslips were removed and washed in cold PBS before mounting them on microscope coverslips to take pictures of living cells with an Imager M2 fluorescence microscope coupled with the AxioCam ICm1 and AxioImager software (Carl Zeiss, Zaventem, Belgium).

#### 4.2.6. Effects of the Extracts on the Cell Cycle and Apoptosis

Concomitant evaluation of the cell cycle and apoptotic process was carried out on the U373 line by flow cytometry using the APO-DIRECT ™ BD Pharmingen kit according to the manufacturer instructions. Briefly, U373 cells were seeded in T25 cm^2^ flasks and allowed to adhere and grow for a minimum of 24 h. Flasks were then treated with MeOH extract (70 μg/mL), EtOAc subfraction (40 μg/mL) or left untreated for 72 h. Supernatants were collected and merged with adherent cells detached by trypsinization. Cells were then fixed with paraformaldehyde 1% in PBS at 4 °C for 1 h, washed with PBS and permeabilized overnight in 70% ice-cold ethanol at −20 °C. The cells were then washed again and incubated with a labeling solution containing the TdT enzyme and the FITC-dUTP substrate for 60 min at 37 °C (TUNEL staining; apoptosis evaluation). Lastly, cells were stained with the propidium iodide solution containing RNAse of the kit for simultaneous cell cycle evaluation. Fluorescence levels of 10,000 events were collected by flow cytometry (Beckman Gallios, Beckman Coulter, Analis, Suarlee, Belgium). The experiment was conducted once in triplicate.

#### 4.2.7. Evaluation of Cellular ROS Species under *E. senegalensis* Treatment

The total intracellular amount of ROS/RNS was evaluated using the diacetylated form of 2′,7′-dichlorodihydrofluorescein diacetate (DCFH-DA; Sigma-Aldrich). DCFH-DA freely enters cells where it is deacetylated into 2′,7′-dichlorodihydrofluorescein (DCFH) by intracellular esterases. DCFH is then oxidized by ROS and RNS (e.g., peroxyl, alkoxy, NO_2_˙, CO_3_˙ and OH˙ radicals and peroxynitrite) to 2′,7′-dichlorofluorescein (DCF), which emits green fluorescence after excitation with a blue laser (488 nm), measured by flow cytometry [122].

The production of mitochondrial ROS, including superoxide anions, was studied using the MitoSox™ Red mitochondrial superoxide probe (Thermofisher, Merelbeke, Belgium). This reagent is a derivative of dihydroethidium bearing a cationic triphenylphosphonium unit that penetrates freely into living cells and then into mitochondria, where it is oxidized by mitochondrial ROS into an ethidium superoxide, which then binds to DNA, producing red fluorescence (after excitation at 488 nm) measured by flow cytometry. Briefly, U373 cells were seeded in 6-well plates. When cells reached 70% to 80% confluence, they were treated with 70 µg/mL of the MeOH extract or 40 µg/mL of the EtOAc extract for 40 h. Cells were washed once with culture medium and incubated separately with each probe prepared beforehand in PBS (MitoSOX: 1 μM for 30 min and DCFHDA: 20 µM for 1 h). Cells were then detached with trypsin, washed once in PBS and resuspended in 250 μL of PBS for flow cytometry analysis (Gallios, Beckmann Coulter, Analis, Suarlée, Belgium). For each sample, data of 10,000 events were recorded. The experiment was conducted once in triplicates.

## 5. Conclusions

This study highlights how the traditional use of *E. senegalensis* may be relevant for its antioxidant properties when considering that the MeOH polar extract is particularly rich in flavonoids and phenolic compounds with potent effects, as observed in DPPH and ABTS assays in particular. The apolar organic extraction with CH_2_Cl_2_ led to decreased antioxidant effects but increased anticancer activity in vitro against a panel of five different cancer models. The EtOAc subfraction retained similar anticancer activity despite lower phenolic and flavonoid content than the MeOH extract. At equipotent concentrations, we observed nevertheless several similar cellular features induced by both MeOH and EtOAc extracts, i.e., cytoplasmic vacuolization that may currently be hypothesized to be related to paraptosis-like induction. Whether this remarkable property is due to one or multiple compounds present in various amounts in those extracts still remains to be deciphered. Indeed, this study provides, for the first time, data supporting the identification of 13 known compounds in *E. senegalensis*, among which several are known to display anticancer and/or antioxidant properties. Nineteen other compounds were also identified. Purification and structure identification or elucidation is ongoing.

## Figures and Tables

**Table 1:** Phytochemical composition of various extracts of *E. Senegalensis*. (+): positive reaction; (−): no reaction; NT: not tested.

Phytochemical Groups	Extracts		
	MeOH	CH_2_Cl_2_	MeOH/CH_2_Cl_2_
Alkaloids	−	−	−
Anthocyanosides	−	−	−
Anthracenosides	+	−	+
Cardiotonic glycosides	−	−	−
Coumarin derivatives	+	−	+
Coumarins	−	+	+
Emodols	+	−	+
Flavonic aglycones	−	+	+
Flavonoids	+	+	+
Saponosides	+	NT	+
Steroidal and triterpene glucosides	+	+	+
Sterols and triterpenes	−	+	+
Tannins	+	NT	+

**Table 2:** Cytotoxic effects (IC_50_) of extracts and primary fractions.

Extracts/Fractions	50% Inhibitory Concentration on Cell Lines				
	(Mean ± SEM) µg/mL				
	U373	MCF-7	A549	SKMEL-28	B16F10
MeOH extract	66 ± 3	38 ± 2	42 ± 1	36 ± 1	33 ± 2
CH_2_Cl_2_/MeOH extract	77 ± 2	41 ± 1	48 ± 1	36 ± 1	33 ± 1
CH_2_Cl_2_ extract	37 ± 2	29 ± 2	19 ± 3	29 ± 1	37 ± 1
EtOAc subfraction	34 ± 1	25 ± 2	19 ± 2	27 ± 2	32 ± 1

**Table 3:** Antioxidant effects and total phenolic and flavonoids content of extracts of *E. senegalensis*. All the experiments were carried out in triplicate and the results are expressed as a mean ± SEM. The results of the DPPH test are given in trolox equivalent antioxidant capacity (TEAC) and those of the ABTS and FRAP tests in mg trolox equivalent/g of dry extract. The total polyphenol content is expressed in mg of gallic acid equivalent/dry extract and that of total flavonoids in mg of quercetin equivalent/dry extract.

Extracts	Antioxidant Capacity			Total Phenolic	Total Flavonoids
	DPPH	ABTS	FRAP		
MeOH	0.56 ± 0.01	0.76 ± 0.02	1.34 ± 0.08	132.54 ± 0.02	101.23 ± 0.08
CH_2_Cl_2_/MeOH	0.67 ± 0.01	1.06 ± 0.03	1.01 ± 0.04	82.01 ± 1.01	47.11 ± 0.52
CH_2_Cl_2_	6.67 ± 0.04	7.92 ± 0.20	12.07 ± 0.04	ND	ND
EtOAc	5.02 ± 0.01	8.23 ± 0.12	10.98 ± 0.05	23 ± 2	19 ± 3

**Table 4:** Substances newly identified from extracts of *E. senegalensis* by LC-MS.

Chemical Group	Substances	Extract Source			Chemical Data			
		MeOH	EtOAc	RT (min)	Formula	Error (ppm)	Measured *m*/*z*	Molecular Species
Triterpene	Uvaol	+		16.149	C_30_H_50_O_2_	1.26	443.3878	[M + H]^+^
Steroids	16:3-Glc-Stigmasterol		+	13.568	C_51_H_82_O_7_	3.87	426.2939	[M + 2Na]^2+^
Steroids	22-Hydroxy-campesterol		+	16.911	C_28_H_48_O_2_	3.99	439.3529	[M + Na]^+^
Steroids	7-Campestenol	+	+	15.344	C_28_H_48_O	2.04	423.3606	[M+ Na]^+^
Steroids	Ergostanol	+		16.141	C_28_H_50_O	4.5	425.3773	[M + Na]^+^
Steroids	Feruloyldihydro-β-sitosterol		+	17.465	C_39_H_60_O_4_	2.41	615.4369	[M + Na]^+^
Steroidal glucoside	Isofucosterol 3-O- 6-O- [Hexadecanoyl-b-D-glucopyranoside]	+	+	12.896	C_51_H_88_O_7_	2.16	445.2887	[M + 2K]^2+^
Fatty acids	9,12-Octadecadienoic acid (Z,Z)	+	+	15.057	C_18_H_32_O_2_	0.19	298.274	[M + NH_4_]^+^
Fatty acids	α-linolinic acid	+	+	14.833	C_18_H_30_O_2_	1.64	279.2314	[M + H]^+^
Fatty acids	Hexadecanoic acid		+	16.223	C_16_H_32_O_2_	0.03	257.2475	[M + H]^+^
Diphenol	1,4-Benzenediol (hydroquinone)	+		5.477	C_6_H_6_O_2_	1.6	128.0704	[M + NH_4_]^+^
Chalcone	2′,4′,6′-Trihydroxy chalcone	+		15.848	C_15_H_12_O_4_	0.25	257.0809	[M + H]^+^

**Table 5:** Substances formerly identified from extracts of *E. senegalensis* by LC-MS.

Chemical Group	Substances	Extract Source			Chemical Data			
		MeOH	EtOAc	RT (min)	Formula	Error (ppm)	Measured *m*/*z*	Molecular Species
Isoflavonoids	2,3-dihydro-auriculatine	+	+	14.615	C_25_H_26_O_6_	0.91	423.1806	[M + H]^+^
Isoflavonoids	Alpinumisoflavone or derrone	+	+	14.689	C_20_H_16_O_5_	1.63	337.1076	[M + H]^+^
Isoflavonoids	Auriculatin or auriculasin	+	+	15.261	C_25_H_24_O_6_	1.75	421.1653	[M + H]^+^
Isoflavonoids	Erysenegalensein N	+		14.64	C_25_H_26_O_7_	1.75	439.1759	[M + H]^+^
Isoflavonoids	Sigmoidin H	+	+	14.96	C_21_H_20_O_5_	0.42	353.1385	[M + H]^+^
Flavonoids	Erythrisenegalone	+	+	15.067	C_25_H_26_O_5_	0.74	407.1856	[M + H]^+^
Flavanone	Senegalensein		+	14.955	C_25_H_28_O_5_	1.1	409.2014	[M + H]^+^
Cinnamate	Erythrinasinate	+		11.532	C_38_H_60_O_4_	4.49	619.4151	[M + K]^+^

**Table 6:** Other putative substances in the EtOAc subfraction (LC-MS data) and whose chemical structure must be determined.

N°	Putative Formula	RT (min)	Measured *m*/*z*	Error (ppm)	Molecular Species
1	C_8_H_4_O_3_	16.764	149.0231	1.48	[M + H]^+^
2	C_8_H_6_O_4_	16.757	167.0341	1.29	[M + H]^+^
3	C_14_H_29_NO	15.246	228.2321	0.4	[M + H]^+^
4	C_16_H_22_O_4_	16.757	279.1592	0.41	[M + H]^+^
5	C_16_H_31_NO	15.4	254.2478	0.16	[M + H]^+^
6	C_16_H_33_NO	15.815	256.2634	0.36	[M + H]^+^
7	C_16_H_34_O_7_	12.67	339.238	0.8	[M + H]^+^
8	C_18_H_32_O	15.945	282.2788	1.21	[M + NH_4_]^+^
9	C_18_H_35_NO	16.217	282.2791	0.15	[M + H]^+^
10	C_18_H_37_NO	16.343	284.2947	0.32	[M + H]^+^
11	C_20_H_42_O_9_	12.956	444.3164	0.69	[M + NH_4_]^+^
12	C_22_H_46_O_10_	13.037	488.343	0.16	[M + NH_4_]^+^
13	C_23_H_48_O_10_	13.25	502.3586	0.05	[M + NH_4_]^+^
14	C_24_H_38_O_4_	16.778	391.284	0.73	[M + H]^+^
15	C_28_H_43_N	17.562	394.3464	1.08	[M + H]^+^
16	C_28_H_46_O_4_	17.581	447.3469	0.03	[M + H]^+^
17	C_36_H_70_N_2_O_2_	15.931	563.5498	2.14	[M + H]^+^
18	C_40_H_69_N_3_O_9_	19.395	736.5099	1.03	[M + H]^+^
19	C_44_H_58_N_2_O_3_	19.388	663.4528	1.18	[M + H]^+^

**Figure A1:**
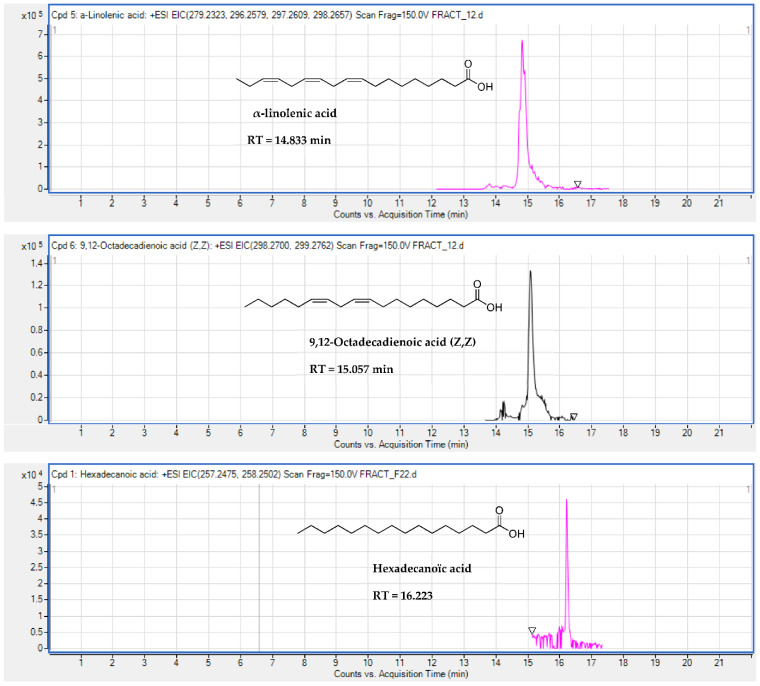

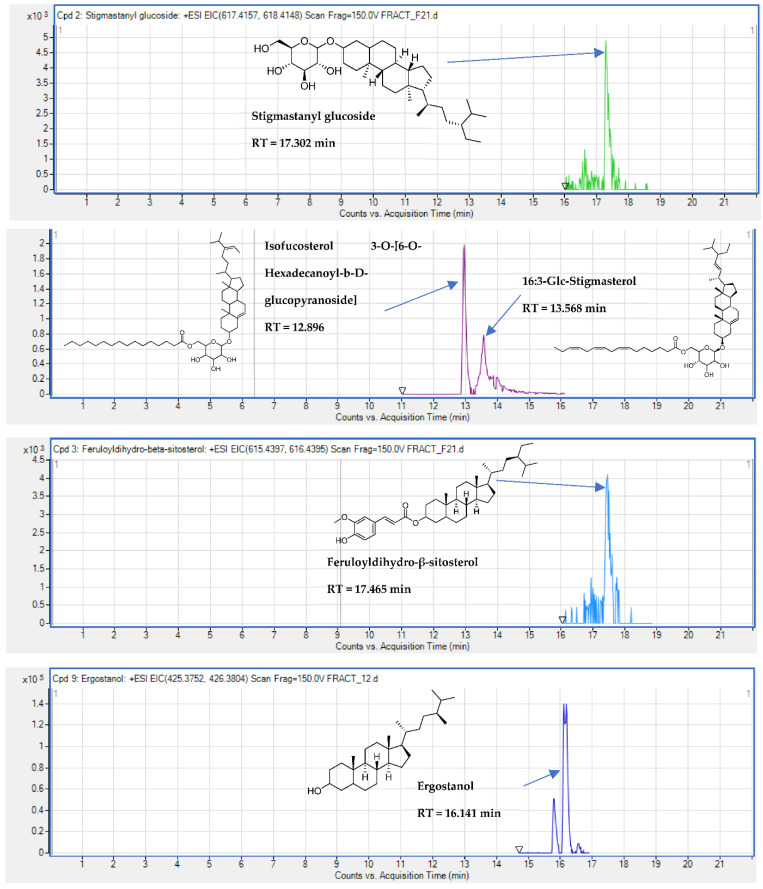

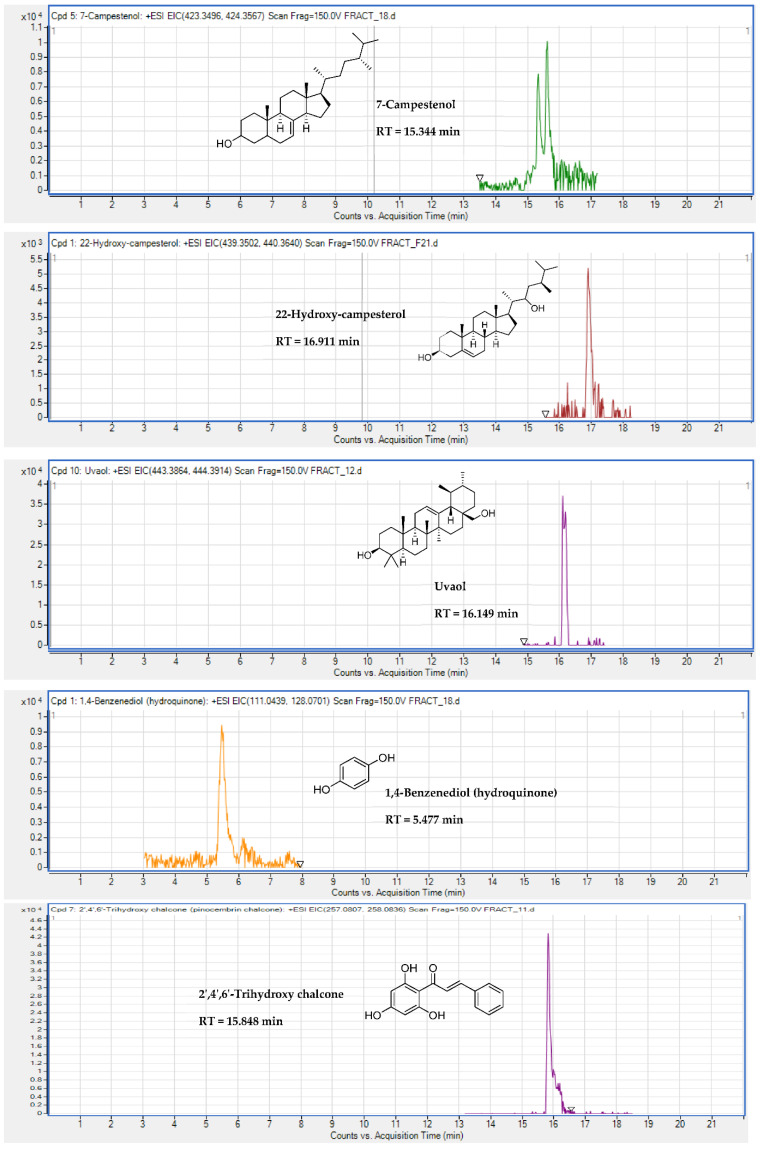
LC-MS chromatogram of newly identified secondary metabolites from *E. senegalensis.* For each compound, an extracted ion chromatogram (EIC) was extracted based on the theoretical values of *m*/*z* of the major adducts and their isotopes.

**Figure A2:**
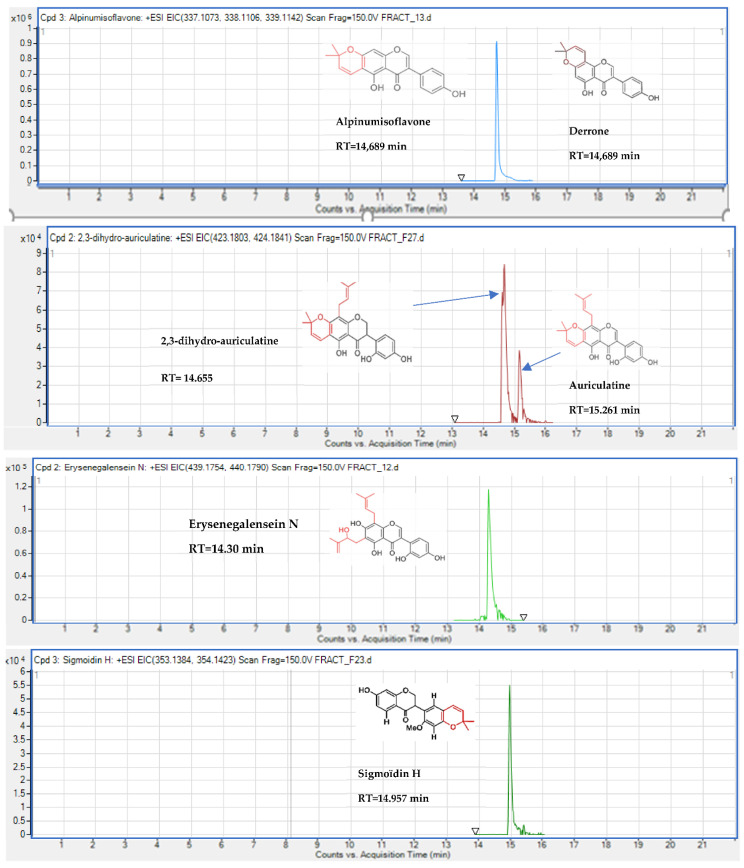

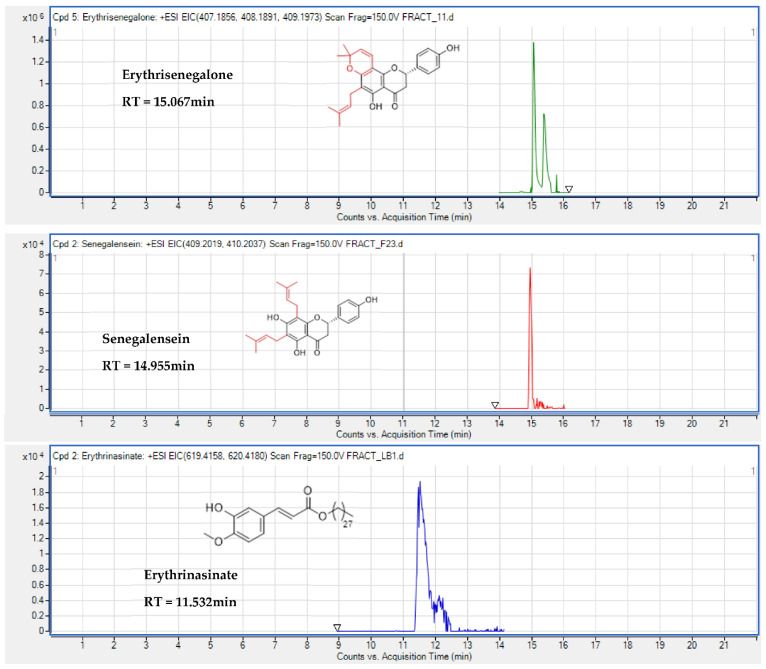
LC-MS chromatogram of formerly identified secondary metabolites from *E. senegalensis*. For each compound, an extracted ion chromatogram (EIC) was extracted based on the theoretical values of *m*/*z* of the major adducts and their isotopes.

## Data Availability

Not applicable.

## Sample Availability

Samples of the compounds (plant materials only) are available from the authors.

## References

[1] Ferlay J., Colombet M., Soerjomataram I., Mathers C., Parkin D.M., Piñeros M., Znaor A., Bray F. (2019). Estimating the Global Cancer Incidence and Mortality in 2018: GLOBOCAN Sources and Methods. Int. J. Cancer.

[2] Newman D.J., Cragg G.M. (2020). Natural Products as Sources of New Drugs over the Nearly Four Decades from 01/1981 to 09/2019. J. Nat. Prod..

[3] Mohammad R.M., Muqbil I., Lowe L., Yedjou C., Hsu H.-Y., Lin L.-T., Siegelin M.D., Fimognari C., Kumar N.B., Dou Q.P. (2015). Broad Targeting of Resistance to Apoptosis in Cancer. Semin. Cancer Biol..

[4] Najafi M., Tavakol S., Zarrabi A., Ashrafizadeh M. (2020). Dual Role of Quercetin in Enhancing the Efficacy of Cisplatin in Chemotherapy and Protection against Its Side Effects: A Review. Arch. Physiol. Biochem..

[5] Wang Z., Li Y.-F., Han X.-Y., Sun Y.-S., Zhang L.-X., Liu W., Liu X.-X., Li W., Liu Y.-Y. (2018). Kidney Protection Effect of Ginsenoside Re and Its Underlying Mechanisms on Cisplatin-Induced Kidney Injury. Cell. Physiol. Biochem. Int. J. Exp. Cell. Physiol. Biochem. Pharmacol..

[6] Bayala B., Nadembega C., Guenné S., Buñay J., Mahoukèdè Zohoncon T., Wendkuuni Djigma F., Yonli A., Baron S., Figueredo G., A Lobaccaro J.-M. (2020). Chemical Composition, Antioxidant and Cytotoxic Activities of *Hyptis Suaveolens* (L.) Poit. Essential Oil on Prostate and Cervical Cancers Cells. Pak. J. Biol. Sci..

[7] Sawadogo W.R., Luo Y., Elkington B., He T.-C., Wang C.-Z., Yuan C.-S. (2020). Cytotoxicity and Preliminary Analysis of the Pro-Apoptotic and Cell Cycle Arrest Effects of Lantana Ukambensis Against Colorectal Cancer Cells. Int. J. Appl. Biol. Pharm. Technol..

[8] Sawadogo W.R., Schumacher M., Teiten M.-H., Dicato M., Diederich M. (2012). Traditional West African Pharmacopeia, Plants and Derived Compounds for Cancer Therapy. Biochem. Pharmacol..

[9] Togola A., Austarheim I., Theïs A., Diallo D., Paulsen B.S. (2008). Ethnopharmacological Uses of Erythrina Senegalensis: A Comparison of Three Areas in Mali, and a Link between Traditional Knowledge and Modern Biological Science. J. Ethnobiol. Ethnomed..

[10] Kuete V., Sandjo L.P., Kwamou G.M.N., Wiench B., Nkengfack A.E., Efferth T. (2014). Activity of Three Cytotoxic Isoflavonoids from Erythrina Excelsa and Erythrina Senegalensis (Neobavaisoflavone, Sigmoidin H and Isoneorautenol) toward Multi-Factorial Drug Resistant Cancer Cells. Phytomed. Int. J. Phytother. Phytopharm..

[11] Bilanda D.C., Bidingha R.À.G., Dzeufiet P.D.D., Fouda Y.B., Ngapout R.F., Tcheutchoua Y., Owona P.E., Wouamba S.C.N., Tatchou L.T., Dimo T. (2020). Antihypertensive and Antidiabetic Activities of Erythrina Senegalensis DC (*Fabaceae*) Stem Bark Aqueous Extract on Diabetic Hypertensive Rats. J. Ethnopharmacol..

[12] Saidu K., Onah J., Orisadipe A., Olusola A., Wambebe C., Gamaniel K. (2000). Antiplasmodial, Analgesic, and Anti-Inflammatory Activities of the Aqueous Extract of the Stem Bark of Erythrina Senegalensis. J. Ethnopharmacol..

[13] Wanjala C.C., Juma B.F., Bojase G., Gashe B.A., Majinda R.R. (2002). Erythrinaline Alkaloids and Antimicrobial Flavonoids from Erythrina Latissima. Planta Med..

[14] Iinuma M., Okawa Y., Tanaka T., Ho F.C., Kobayashi Y., Miyauchi K.I. (1994). Anti-Oral Microbial Activity of Isoflavonoids in Root Bark of Ormosia Monosperma. Phytochemistry.

[15] Meragelman K.M., McKee T.C., Boyd M.R. (2001). Anti-HIV Prenylated Flavonoids from Monotes Africanus. J. Nat. Prod..

[16] Lyddiard J.R.A., Whitfield P.J., Bartlett A. (2002). Antischistosomal Bioactivity of Isoflavonoids from Millettia Thonningii (Leguminosae). J. Parasitol..

[17] Dramane S., Koné M., Kamanzi K. (2010). Evaluation of Antimicrobial and Free Radical Scavenging Activities of Some Bioactif Taxons from Côte d’Ivoire. Eur. J. Sci. Res..

[18] Niwa T., Murakami K., Ohtake T., Etoh H., Shimizu A., Shimizu Y., Kato Y., Tanaka H. (2002). Peroxynitrite Scavenging Activities of Aromatic Compounds Isolated from Konnyaku, Amorphophallus Konjac K.Koch. Biosci. Biotechnol. Biochem..

[19] Togola A., Hedding B., Theis A., Wangensteen H., Rise F., Smestad Paulsen B., Diallo D., Egil Malterud K. (2009). 15-Lipoxygenase Inhibitory Effects of Prenylated Flavonoids from Erythrina Senegalensis. Planta Med..

[20] Oh W.K., Lee C.-H., Seo J.H., Chung M.Y., Cui L., Fomum Z.T., Kang J.S., Lee H.S. (2009). Diacylglycerol Acyltransferase-Inhibitory Compounds from Erythrina Senegalensis. Arch. Pharm. Res..

[21] Majeed I., Rizwan K., Ashar A., Rasheed T., Amarowicz R., Kausar H., Zia-Ul-Haq M., Marceanu L.G. (2021). A Comprehensive Review of the Ethnotraditional Uses and Biological and Pharmacological Potential of the Genus Mimosa. Int. J. Mol. Sci..

[22] Rizwan K., Majeed I., Bilal M., Rasheed T., Shakeel A., Iqbal S. (2022). Phytochemistry and Diverse Pharmacology of Genus Mimosa: A Review. Biomolecules.

[23] Fofana S., Ouédraogo M., Esposito R.C., Ouedraogo W.P., Delporte C., Van Antwerpen P., Mathieu V., Guissou I.P. (2021). Systematic Review of Potential Anticancerous Activities of Erythrina Senegalensis DC (*Fabaceae*). Plants.

[24] Zhou W., Zeng X., Wu X. (2020). Effect of Oleanolic Acid on Apoptosis and Autophagy of SMMC-7721 Hepatoma Cells. Med. Sci. Monit. Int. Med. J. Exp. Clin. Res..

[25] Gao C., Li X., Yu S., Liang L. (2019). Inhibition of Cancer Cell Growth by Oleanolic Acid in Multidrug Resistant Liver Carcinoma Is Mediated via Suppression of Cancer Cell Migration and Invasion, Mitochondrial Apoptosis, G2/M Cell Cycle Arrest and Deactivation of JNK/P38 Signalling Pathway. J. BUON.

[26] Juan M.E., Wenzel U., Daniel H., Planas J.M. (2008). Erythrodiol, a Natural Triterpenoid from Olives, Has Antiproliferative and Apoptotic Activity in HT-29 Human Adenocarcinoma Cells. Mol. Nutr. Food Res..

[27] Namkoong S., Kim T.-J., Jang I.-S., Kang K.-W., Oh W.-K., Park J. (2011). Alpinumisoflavone Induces Apoptosis and Suppresses Extracellular Signal-Regulated Kinases/Mitogen Activated Protein Kinase and Nuclear Factor-ΚB Pathways in Lung Tumor Cells. Biol. Pharm. Bull..

[28] Li D., Li X., Li G., Meng Y., Jin Y., Shang S., Li Y. (2019). Alpinumisoflavone Causes DNA Damage in Colorectal Cancer Cells via Blocking DNA Repair Mediated by RAD51. Life Sci..

[29] Zhang Y., Yang H., Sun M., He T., Liu Y., Yang X., Shi X., Liu X. (2020). Alpinumisoflavone Suppresses Hepatocellular Carcinoma Cell Growth and Metastasis via NLRP3 Inflammasome-Mediated Pyroptosis. Pharmacol. Rep..

[30] Gao M., Chang Y., Wang X., Ban C., Zhang F. (2017). Reduction of COX-2 through Modulating MiR-124/SPHK1 Axis Contributes to the Antimetastatic Effect of Alpinumisoflavone in Melanoma. Am. J. Transl. Res..

[31] Hoang N.T.M., Phuong T.T., Nguyen T.T.N., Tran Y.T.H., Nguyen A.T.N., Nguyen T.L., Bui K.T.V. (2016). In Vitro Characterization of Derrone as an Aurora Kinase Inhibitor. Biol. Pharm. Bull..

[32] Kang M.-J., Kim S.-Y., Kwon E.-B., Jo Y.H., Lee M.K., Lee H.-S., Moon D.-O., Kim M.-O. (2019). Derrone Induces Autophagic Cell Death through Induction of ROS and ERK in A549 Cells. PLoS ONE.

[33] Ito C., Murata T., Itoigawa M., Nakao K., Kumagai M., Kaneda N., Furukawa H. (2006). Induction of Apoptosis by Isoflavonoids from the Leaves of Millettia Taiwaniana in Human Leukemia HL-60 Cells. Planta Med..

[34] Min H.-Y., Jung Y., Park K.H., Oh W.K., Lee H.-Y. (2018). Erybraedin A Is a Potential Src Inhibitor That Blocks the Adhesion and Viability of Non-Small-Cell Lung Cancer Cells. Biochem. Biophys. Res. Commun..

[35] Maurich T., Iorio M., Chimenti D., Turchi G. (2006). Erybraedin C and Bitucarpin A, Two Structurally Related Pterocarpans Purified from Bituminaria Bituminosa, Induced Apoptosis in Human Colon Adenocarcinoma Cell Lines MMR- and P53-Proficient and -Deficient in a Dose-, Time-, and Structure-Dependent Fashion. Chem. Biol. Interact..

[36] Rukachaisirikul T., Saekee A., Tharibun C., Watkuolham S., Suksamrarn A. (2007). Biological Activities of the Chemical Constituents of Erythrina Stricta and Erythrina Subumbrans. Arch. Pharm. Res..

[37] Wätjen W., Kulawik A., Suckow-Schnitker A.K., Chovolou Y., Rohrig R., Ruhl S., Kampkötter A., Addae-Kyereme J., Wright C.W., Passreiter C.M. (2007). Pterocarpans Phaseollin and Neorautenol Isolated from Erythrina Addisoniae Induce Apoptotic Cell Death Accompanied by Inhibition of ERK Phosphorylation. Toxicology.

[38] Nie H., Wang Y., Qin Y., Gong X.-G. (2016). Oleanolic Acid Induces Autophagic Death in Human Gastric Cancer Cells In Vitro and In Vivo. Cell Biol. Int..

[39] Hosny S., Sahyon H., Youssef M., Negm A. (2021). Oleanolic Acid Suppressed DMBA-Induced Liver Carcinogenesis through Induction of Mitochondrial-Mediated Apoptosis and Autophagy. Nutr. Cancer.

[40] Liu J., Zheng L., Ma L., Wang B., Zhao Y., Wu N., Liu G., Lin X. (2014). Oleanolic Acid Inhibits Proliferation and Invasiveness of Kras-Transformed Cells via Autophagy. J. Nutr. Biochem..

[41] Sheu Y.-W., Chiang L.-C., Chen I.-S., Chen Y.-C., Tsai I.-L. (2005). Cytotoxic Flavonoids and New Chromenes from Ficus Formosana f. Formosana. Planta Med..

[42] Ukiya M., Akihisa T., Tokuda H., Suzuki H., Mukainaka T., Ichiishi E., Yasukawa K., Kasahara Y., Nishino H. (2002). Constituents of Compositae Plants III. Anti-Tumor Promoting Effects and Cytotoxic Activity against Human Cancer Cell Lines of Triterpene Diols and Triols from Edible Chrysanthemum Flowers. Cancer Lett..

[43] Ito A., Chai H.-B., Kardono L.B.S., Setowati F.M., Afriastini J.J., Riswan S., Farnsworth N.R., Cordell G.A., Pezzuto J.M., Swanson S.M. (2004). Saponins from the Bark of Nephelium Maingayi. J. Nat. Prod..

[44] Ateba S.B., Mvondo M.A., Djiogue S., Zingué S., Krenn L., Njamen D. (2019). A Pharmacological Overview of Alpinumisoflavone, a Natural Prenylated Isoflavonoid. Front. Pharmacol..

[45] Tjahjandarie T.S., Tanjung M. (2015). Phenolic Compounds from the Stem Bark of Erythrina Orientalis and Their Cytotoxic and Antioxidant Activities. Pharma Chem..

[46] Akter K., Barnes E.C., Loa-Kum-Cheung W.L., Yin P., Kichu M., Brophy J.J., Barrow R.A., Imchen I., Vemulpad S.R., Jamie J.F. (2016). Antimicrobial and Antioxidant Activity and Chemical Characterisation of Erythrina Stricta Roxb. (*Fabaceae*). J. Ethnopharmacol..

[47] Fu G., Li W., Huang X., Zhang R., Tian K., Hou S., Li Y. (2018). Antioxidant and Alpha-Glucosidase Inhibitory Activities of Isoflavonoids from the Rhizomes of Ficus Tikoua Bur. Nat. Prod. Res..

[48] Bai X., Lai T., Zhou T., Li Y., Li X., Zhang H. (2018). In Vitro Antioxidant Activities of Phenols and Oleanolic Acid from Mango Peel and Their Cytotoxic Effect on A549 Cell Line. Mol. J. Synth. Chem. Nat. Prod. Chem..

[49] Song Y., Wang J., Zhang C., Yu Y., Cai H. (2021). Proapoptotic Protein Smac Mediates Apoptosis in Ovarian Cancer Cells When Treated with Carpachromene. Arch. Med. Sci..

[50] Issarachot P., Sangkaew W., Sianglum W., Saeloh D., Limsuwan S., Voravuthikunchai S.P., Joycharat N. (2021). α-Glucosidase Inhibitory, Antibacterial, and Antioxidant Activities of Natural Substances from the Wood of Derris Reticulata Craib. Nat. Prod. Res..

[51] Allouche Y., Beltrán G., Gaforio J.J., Uceda M., Mesa M.D. (2010). Antioxidant and Antiatherogenic Activities of Pentacyclic Triterpenic Diols and Acids. Food Chem. Toxicol..

[52] Allouche Y., Warleta F., Campos M., Sánchez-Quesada C., Uceda M., Beltrán G., Gaforio J.J. (2011). Antioxidant, Antiproliferative, and pro-Apoptotic Capacities of Pentacyclic Triterpenes Found in the Skin of Olives on MCF-7 Human Breast Cancer Cells and Their Effects on DNA Damage. J. Agric. Food Chem..

[53] Martín R., Ibeas E., Carvalho-Tavares J., Hernández M., Ruiz-Gutierrez V., Nieto M.L. (2009). Natural Triterpenic Diols Promote Apoptosis in Astrocytoma Cells through ROS-Mediated Mitochondrial Depolarization and JNK Activation. PLoS ONE.

[54] Klaunig J.E. (2018). Oxidative Stress and Cancer 2018. Curr. Pharm. Des..

[55] Mileo A.M., Miccadei S. (2016). Polyphenols as Modulator of Oxidative Stress in Cancer Disease: New Therapeutic Strategies. Oxid. Med. Cell. Longev..

[56] Saha S.K., Lee S.B., Won J., Choi H.Y., Kim K., Yang G.-M., Dayem A.A., Cho S.-G. (2017). Correlation between Oxidative Stress, Nutrition, and Cancer Initiation. Int. J. Mol. Sci..

[57] Pilco-Ferreto N., Calaf G.M. (2016). Influence of Doxorubicin on Apoptosis and Oxidative Stress in Breast Cancer Cell Lines. Int. J. Oncol..

[58] Fofana S., Gnoula C., Ouedraogo Moussa M., Palé E., Nebié R.H., Nikiema J.-B., Guissou I.P., Simporé J. (2016). DPPH Radical Scavenging and Lipoxygenase Inhibitory Effects in Extracts from Erythrina Senegalensis (*Fabaceae*) DC. Afr. J. Pharm. Pharmacol..

[59] Ivanov A.V., Bartosch B., Isaguliants M.G. (2017). Oxidative Stress in Infection and Consequent Disease. Oxid. Med. Cell. Longev..

[60] Vasquez M., Zuniga M., Rodriguez A. (2021). Oxidative Stress and Pathogenesis in Malaria. Front. Cell. Infect. Microbiol..

[61] Nacoulma O.G. (1996). Plantes Médicinales et Pratiques Médicales Traditionnelles Au Burkina Faso: Cas Du Plateau Central. Fac. Sci. Tech. Univ. Ouagadougou.

[62] Wandji J., Nkengfack A.E., Fomum Z.T., Ubillas R., Killday K.B., Tempesta M.S. (1990). A New Prenylated Isoflavone and Long Chain Esters from Two Erythrina Species. J. Nat. Prod..

[63] Wandji J., Fomum Z.T., Tillequin F., Baudouin G., Koch M. (1994). Epoxyisoflavones from Erythrina Senegalensis. Phytochemistry.

[64] Wandji J., Fomum Z.T., Tillequin F., Libot F., Koch M. (1995). Erysenegalenseins B and C, Two New Prenylated Isoflavanones from Erythrina Senegalensis. J. Nat. Prod..

[65] Wandji J., Fomum Z.T., Tillequin F., Skaltsounis A.L., Koch M. (1994). Erysenegalenseins H and I: Two New Isoflavones from Erythrina Senegalensis1. Planta Med..

[66] Fomum Z.T., Ayafor J.F., Wandji J. (1985). Erythrisenegalone, a Prenylated-Flavanone from Erythrina Senegalensis. Phytochemistry.

[67] Wandji J., Awanchiri S., Fomum Z.T., Tillequin F., Libot F. (1995). Isoflavones and Alkaloids from the Stem Bark and Seeds of Erythrina Senegalensis. Phytochemistry.

[68] Oh W.K., Lee H.S., Ahn S.C., Ahn J.S., Mbafor J.T., Wandji J., Fomum Z.T., Chang H.K., Kim Y.H. (1999). Prenylated Isoflavonoids from Erythrina Senegalensis. Phytochemistry.

[69] Taylor R.B., Corley D.G., Tempesta M.S., Fomum Z.T., Ayafor J.F., Wandji J., Ifeadike P.N. (1986). 2,3-Dihydroauriculatin, a New Prenylated Isoflavanone from Erythrina Senegalensis. Application of the Selective Inept Technique. J. Nat. Prod..

[70] Kuete V., Mbaveng A.T., Nono E.C.N., Simo C.C., Zeino M., Nkengfack A.E., Efferth T. (2016). Cytotoxicity of Seven Naturally Occurring Phenolic Compounds towards Multi-Factorial Drug-Resistant Cancer Cells. Phytomed. Int. J. Phytother. Phytopharm..

[71] Jaramillo-Rangel G., Chávez-Briones M.-L., Niderhauser-García A., Ortega-Martínez M. (2020). Toxicity and Anticancer Potential of Karwinskia: A Review. Molecules.

[72] Semwal R.B., Semwal D.K., Combrinck S., Viljoen A. (2021). Emodin—A Natural Anthraquinone Derivative with Diverse Pharmacological Activities. Phytochemistry.

[73] Cui Y., Chen L.-J., Huang T., Ying J.-Q., Li J. (2020). The Pharmacology, Toxicology and Therapeutic Potential of Anthraquinone Derivative Emodin. Chin. J. Nat. Med..

[74] Gnoula C., Mégalizzi V., De Nève N., Sauvage S., Ribaucour F., Guissou P., Duez P., Dubois J., Ingrassia L., Lefranc F. (2008). Balanitin-6 and -7: Diosgenyl Saponins Isolated from Balanites Aegyptiaca Del. Display Significant Anti-Tumor Activity In Vitro and In Vivo. Int. J. Oncol..

[75] Sobolewska D., Michalska K., Podolak I., Grabowska K. (2016). Steroidal Saponins from the Genus Allium. Phytochem. Rev. Proc. Phytochem. Soc. Eur..

[76] Grabowska K., Podolak I., Galanty A., Żmudzki P., Koczurkiewicz P., Piska K., Pękala E., Janeczko Z. (2017). Two New Triterpenoid Saponins from the Leaves of Impatiens Parviflora DC. and Their Cytotoxic Activity. Ind. Crops Prod..

[77] Moilanen J., Karonen M., Tähtinen P., Jacquet R., Quideau S., Salminen J.-P. (2016). Biological Activity of Ellagitannins: Effects as Anti-Oxidants, pro-Oxidants and Metal Chelators. Phytochemistry.

[78] Yang L., Xian D., Xiong X., Lai R., Song J., Zhong J. (2018). Proanthocyanidins against Oxidative Stress: From Molecular Mechanisms to Clinical Applications. BioMed Res. Int..

[79] Cao J., Han J., Xiao H., Qiao J., Han M. (2016). Effect of Tea Polyphenol Compounds on Anticancer Drugs in Terms of Anti-Tumor Activity, Toxicology, and Pharmacokinetics. Nutrients.

[80] Bouhenna M.M., Mameri N., Pérez M.V., Talhi O., Bachari K., Silva A.M.S., Luyten W. (2018). Anticancer Activity Study of Chromone and Coumarin Hybrids Using Electrical Impedance Spectroscopy. Anticancer Agents Med. Chem..

[81] Caro A.A., Davis A., Fobare S., Horan N., Ryan C., Schwab C. (2019). Antioxidant and Pro-Oxidant Mechanisms of (+) Catechin in Microsomal CYP2E1-Dependent Oxidative Stress. Toxicol. In Vitro.

[82] Kopustinskiene D.M., Jakstas V., Savickas A., Bernatoniene J. (2020). Flavonoids as Anticancer Agents. Nutrients.

[83] Sahebkar A. (2015). Dual Effect of Curcumin in Preventing Atherosclerosis: The Potential Role of pro-Oxidant-Antioxidant Mechanisms. Nat. Prod. Res..

[84] Liu Y., Levine B. (2015). Autosis and Autophagic Cell Death: The Dark Side of Autophagy. Cell Death Differ..

[85] Bury M., Girault A., Mégalizzi V., Spiegl-Kreinecker S., Mathieu V., Berger W., Evidente A., Kornienko A., Gailly P., Vandier C. (2013). Ophiobolin A Induces Paraptosis-like Cell Death in Human Glioblastoma Cells by Decreasing BKCa Channel Activity. Cell Death Dis..

[86] Mao L., Liu H., Liu H., Bian Z., Zhang Q., Liao W., Sun S. (2020). Preparation of warangalone-loaded liposomes and its inhibitory effect on breast cancer cells. J. South. Med. Univ..

[87] Tchokouaha R.F., Alexi X., Chosson E., Besson T., Skaltsounis A.-L., Seguin E., Alexis M.N., Wandji J. (2010). Erymildbraedin A and B, Two Novel Cytotoxic Dimethylpyrano-Isoflavones from the Stem Bark of Erythrina Mildbraedii: Evaluation of Their Activity toward Endocrine Cancer Cells. J. Enzyme Inhib. Med. Chem..

[88] Nkengfack A.E., Azebaze A.G., Waffo A.K., Fomum Z.T., Meyer M., van Heerden F.R. (2001). Cytotoxic Isoflavones from Erythrina Indica. Phytochemistry.

[89] Zhang B., Fan X., Wang Z., Zhu W., Li J. (2017). Alpinumisoflavone Radiosensitizes Esophageal Squamous Cell Carcinoma through Inducing Apoptosis and Cell Cycle Arrest. Biomed. Pharmacother..

[90] Desta Z.Y., Sewald N., Majinda R.R. (2016). Cytotoxic Flavonoids from Erythrina Caffra Thunb. Bull. Chem. Soc. Ethiop..

[91] Byeon S.E., Yi Y.-S., Lee J., Yang W.S., Kim J.H., Kim J., Hong S., Kim J.-H., Cho J.Y. (2018). Hydroquinone Exhibits In Vitro and In Vivo Anti-Cancer Activity in Cancer Cells and Mice. Int. J. Mol. Sci..

[92] Sunassee S., Davies-Coleman M. (2012). Cytotoxic and Antioxidant Marine Prenylated Quinones and Hydroquinones. Nat. Prod. Rep..

[93] Chang M.-C., Chang B.-E., Pan Y.-H., Lin B.-R., Lian Y.-C., Lee M.-S., Yeung S.-Y., Lin L.-D., Jeng J.-H. (2019). Antiplatelet, Antioxidative, and Anti-Inflammatory Effects of Hydroquinone. J. Cell. Physiol..

[94] Sofi M.S., Sateesh M.K., Bashir M., Ganie M.A., Nabi S. (2018). Chemopreventive and Anti-Breast Cancer Activity of Compounds Isolated from Leaves of *Abrus Precatorius* L.. 3 Biotech.

[95] Bae H., Song G., Lim W. (2020). Stigmasterol Causes Ovarian Cancer Cell Apoptosis by Inducing Endoplasmic Reticulum and Mitochondrial Dysfunction. Pharmaceutics.

[96] Liao H., Zhu D., Bai M., Chen H., Yan S., Yu J., Zhu H., Zheng W., Fan G. (2020). Stigmasterol Sensitizes Endometrial Cancer Cells to Chemotherapy by Repressing Nrf2 Signal Pathway. Cancer Cell Int..

[97] Sheu J.H., Wang G.H., Sung P.J., Duh C.Y. (1999). New Cytotoxic Oxygenated Fucosterols from the Brown Alga Turbinaria Conoides. J. Nat. Prod..

[98] Sheu J.H., Wang G.H., Sung P.J., Chiu Y.H., Duh C.Y. (1997). Cytotoxic Sterols from the Formosan Brown Alga Turbinaria Ornata. Planta Med..

[99] Pacheco B.S., Dos Santos M.A.Z., Schultze E., Martins R.M., Lund R.G., Seixas F.K., Colepicolo P., Collares T., Paula F.R., De Pereira C.M.P. (2018). Cytotoxic Activity of Fatty Acids from Antarctic Macroalgae on the Growth of Human Breast Cancer Cells. Front. Bioeng. Biotechnol..

[100] Jiang H., Li J., Chen A., Li Y., Xia M., Guo P., Yao S., Chen S. (2018). Fucosterol Exhibits Selective Antitumor Anticancer Activity against HeLa Human Cervical Cell Line by Inducing Mitochondrial Mediated Apoptosis, Cell Cycle Migration Inhibition and Downregulation of m-TOR/PI3K/Akt Signalling Pathway. Oncol. Lett..

[101] Shen T., Zhang L., Wang Y.-Y., Fan P.-H., Wang X.-N., Lin Z.-M., Lou H.-X. (2012). Steroids from Commiphora Mukul Display Antiproliferative Effect against Human Prostate Cancer PC3 Cells via Induction of Apoptosis. Bioorg. Med. Chem. Lett..

[102] Feng M.-T., Wang T., Liu A.-H., Li J., Yao L.-G., Wang B., Guo Y.-W., Mao S.-C. (2018). PTP1B Inhibitory and Cytotoxic C-24 Epimers of Δ28-24-Hydroxy Stigmastane-Type Steroids from the Brown Alga Dictyopteris Undulata Holmes. Phytochemistry.

[103] Bonel-Pérez G.C., Pérez-Jiménez A., Gris-Cárdenas I., Parra-Pérez A.M., Lupiáñez J.A., Reyes-Zurita F.J., Siles E., Csuk R., Peragón J., Rufino-Palomares E.E. (2020). Antiproliferative and Pro-Apoptotic Effect of Uvaol in Human Hepatocarcinoma HepG2 Cells by Affecting G0/G1 Cell Cycle Arrest, ROS Production and AKT/PI3K Signaling Pathway. Molecules.

[104] Bano Z., Begum S., Ali S.S., Kiran Z., Siddiqui B.S., Ahmed A., Khawaja S., Fatima F., Jabeen A. (2021). Phytochemicals from *Carissa Carandas* with Potent Cytotoxic and Anti-Inflammatory Activities. Nat. Prod. Res..

[105] Sperandio S., de Belle I., Bredesen D.E. (2000). An Alternative, Nonapoptotic Form of Programmed Cell Death. Proc. Natl. Acad. Sci. USA.

[106] Yoon M.J., Lee A.R., Jeong S.A., Kim Y.-S., Kim J.Y., Kwon Y.-J., Choi K.S. (2014). Release of Ca^2+^ from the Endoplasmic Reticulum and Its Subsequent Influx into Mitochondria Trigger Celastrol-Induced Paraptosis in Cancer Cells. Oncotarget.

[107] Pistritto G., Trisciuoglio D., Ceci C., Garufi A., D’Orazi G. (2016). Apoptosis as Anticancer Mechanism: Function and Dysfunction of Its Modulators and Targeted Therapeutic Strategies. Aging.

[108] Wang Y., Wen X., Zhang N., Wang L., Hao D., Jiang X., He G. (2019). Small-Molecule Compounds Target Paraptosis to Improve Cancer Therapy. Biomed. Pharmacother..

[109] Butterfield D.A., Boyd-Kimball D. (2018). Oxidative Stress, Amyloid-β Peptide, and Altered Key Molecular Pathways in the Pathogenesis and Progression of Alzheimer’s Disease. J. Alzheimers Dis..

[110] Sies H., Berndt C., Jones D.P. (2017). Oxidative Stress. Annu. Rev. Biochem..

[111] Ahotupa M. (2017). Oxidized Lipoprotein Lipids and Atherosclerosis. Free Radic. Res..

[112] Senoner T., Dichtl W. (2019). Oxidative Stress in Cardiovascular Diseases: Still a Therapeutic Target?. Nutrients.

[113] Jakubczyk K., Dec K., Kałduńska J., Kawczuga D., Kochman J., Janda K. (2020). Reactive Oxygen Species—Sources, Functions, Oxidative Damage. Pol. Merkur. Lek. Organ Pol. Tow. Lek..

[114] Ciulei I. (1982). Methodology for Analysis of Vegetable Drugs. Practical Manual on the Industrial Utilisation of Medicinal and Aromatic Plants..

[115] Meda A., Lamien C.E., Romito M., Millogo J., Nacoulma O.G. (2005). Determination of the Total Phenolic, Flavonoid and Proline Contents in Burkina Fasan Honey, as Well as Their Radical Scavenging Activity. Food Chem..

[116] Arvouet-Grand A., Vennat B., Pourrat A., Legret P. (1994). Standardization of propolis extract and identification of principal constituents. J. Pharm. Belg..

[117] Xiao F., Xu T., Lu B., Liu R. (2020). Guidelines for Antioxidant Assays for Food Components. Food Front..

[118] Nenadis N., Wang L.-F., Tsimidou M., Zhang H.-Y. (2004). Estimation of Scavenging Activity of Phenolic Compounds Using the ABTS(*+) Assay. J. Agric. Food Chem..

[119] Benzie I.F.F., Strain J.J. (1996). The Ferric Reducing Ability of Plasma (FRAP) as a Measure of “Antioxidant Power”: The FRAP Assay. Anal. Biochem..

[120] Bunea A., Rugină D., Sconţa Z., Pop R.M., Pintea A., Socaciu C., Tăbăran F., Grootaert C., Struijs K., VanCamp J. (2013). Anthocyanin Determination in Blueberry Extracts from Various Cultivars and Their Antiproliferative and Apoptotic Properties in B16-F10 Metastatic Murine Melanoma Cells. Phytochemistry.

[121] Colin M., Delporte C., Janky R., Lechon A.-S., Renard G., Van Antwerpen P., Maltese W.A., Mathieu V. (2019). Dysregulation of Macropinocytosis Processes in Glioblastomas May Be Exploited to Increase Intracellular Anti-Cancer Drug Levels: The Example of Temozolomide. Cancers.

[122] Eruslanov E., Kusmartsev S. (2010). Identification of ROS Using Oxidized DCFDA and Flow-Cytometry. Advanced Protocols in Oxidative Stress II.

